# Changes in behavior and quality of life in German young children during the COVID-19 pandemic—results from the COVID kids bavaria study

**DOI:** 10.3389/fped.2023.1135415

**Published:** 2023-05-09

**Authors:** Hannah Schillok, Michaela Coenen, Eva A. Rehfuess, Pia H. Kuhlmann, Stefan Matl, Hannah Kindermann, Nicole Maison, Jana Eckert, Ulrich von Both, Uta Behrends, Michael C. Frühwald, Antje Neubert, Joachim Woelfle, Michael Melter, Johannes Liese, Johannes Hübner, Christoph Klein, Anna Kern, Caroline Jung-Sievers

**Affiliations:** ^1^ Institute for Medical Information Processing, Biometry, and Epidemiology (IBE), Chair of Public Health and Health Services Research, LMU Munich, Munich, Germany; ^2^ Pettenkofer School of Public Health, Munich, Germany; ^3^ Department of Pediatrics, Dr. von Hauner Children’s Hospital, University Hospital LMU Munich, Munich, Germany; ^4^ German Center for Lung Research (DZL), Munich, Germany; ^5^ Institute for Asthma- and Allergy Prevention (IAP), Helmholtz Center Munich, German Research Center for Environmental Health (GmbH), Munich, Germany; ^6^ German Center for Infection Research (DZIF), Partner Site Munich, Munich, Germany; ^7^ Department of Pediatrics, Kinderklinik München Schwabing, StKM GmbH und Klinikum Rechts der Isar, Technische Universität München, Munich, Germany; ^8^ Paediatric and Adolescent Medicine University Hospital Augsburg, Augsburg, Germany; ^9^ Department of Paediatric and Adolescent Medicine, Erlangen University Hospital, Erlangen, Germany; ^10^ University Children’s Hospital Regensburg (KUNO), Regensburg, Germany; ^11^ Department of Pediatrics, University Hospital Würzburg, Würzburg, Germany

**Keywords:** COVID-19, pandemic, mental health—related quality of life, behavior change, health-related quality of life (HRQL), health inequalities in children

## Abstract

**Introduction:**

The COVID-19 pandemic with its containment measures such as closures of schools and daycare facilities led to numerous restrictions in daily life, putting developmental opportunities and health-related quality of life in children at risk. However, studies show that not every family was impacted equally by the pandemic and that this exceptional health and societal situation reinforced pre-existing health inequalities among the vulnerable. Our study aimed at analyzing changes in behavior and health-related quality of life of children attending elementary schools and daycare facilities in Bavaria, Germany in spring 2021. We also sought to identify associated factors contributing to inequalities in quality of life.

**Methods:**

Data from a multi-center, open cohort study (“COVID Kids Bavaria”) conducted in 101 childcare facilities and 69 elementary schools across all electoral districts of Bavaria were analyzed. Children attending these educational settings (aged 3-10 years) were eligible for participation in a survey on changes in behavior and health-related quality of life. The KINDL^R^ questionnaire (based on children’s self-report and parental report) was administered about one year after the onset of the pandemic (spring 2021). Descriptive and logistic regression analyses and comparisons to pre-pandemic KiGGS (German Health Interview and Examination Survey for Children and Adolescents) data were undertaken.

**Results:**

Among respondents, a high percentage of parents reported changes in their children's eating and sleeping behavior, sports and outdoor activities as well as altered screen time. Health-related quality of life in KINDL^R^ analyses compared to pre-pandemic population averages were lower in all age groups (for 3–6-year-old KINDL^R^-total score: COVID Kids Bavaria MD 74.78 ± 10.57 vs KiGGS data 80.0 ± 8.1; 7-10 years-old KINDL^R^-total score: COVID Kids Bavaria MD 73.88 ± 12.03 vs KiGGS data 79.30 ± 9.0). No significant differences were detected with regard to associated factors, namely type of institution, sex of the child, migration background, household size and parental education.

**Conclusion:**

These findings suggest a relevant impact of the COVID-19 pandemic on children’s behavior and health-related quality of life one year after the onset of the pandemic. Further analyses in large-scale longitudinal studies are needed to determine the effects of specific pandemic or crisis associated factors contributing to health inequalities.

## 1. Introduction

The global spread of the SARS-CoV-2 virus can be seen as a worldwide stress test for populations around the world, but especially for vulnerable groups. Even if children are only mildly affected by a SARS-CoV-2 infection in general, they represent a particularly vulnerable group with regards to the consequences of mitigation measures taken against the spread of the virus during different stages of the pandemic (1, 2). Being in a critical developmental phase, they were at times deprived of educational opportunities and social contacts by the closure or reduced services of schools and childcare institutions. At the same time, they were confronted with stressors such as isolation, stressed parents due to potentially insecure social and economic situations, and loss of daily structure through restricted leisure activities. In addition, they may have had less resilience capacity due to their lack of life experience compared to adults.

During the pandemic, the mental health of children has been compromised as demonstrated by many international studies and systematic reviews (3–10). A meta-analysis of studies on the prevalence of depressive and anxiety symptoms in children and adolescents during the pandemic demonstrates a two-fold increase in these symptoms (4). Certain groups, such as children with preexisting (chronic) physical or mental health conditions and lower socioeconomic family resources suffer disproportionately (11).

After a rapid increase in the first phase of the pandemic, stress and psychosocial symptoms have remained at high and relatively stable levels throughout the pandemic. Ravens-Sieberer et al. reported in the COPSY (longitudinal COVID-19 and Psychological Health) study that two-thirds of children and adolescents were highly burdened by the COVID-19 pandemic with lower health-related quality of life (QoL), more mental health problems and worsened behavior compared to pre-pandemic times. However, levels of mental health problems between the first wave in spring 2020 and the second wave in winter 2020/2021 did not differ significantly (12–16). Analyses of the German serial cross-sectional “Corona Snapshot Monitoring” (COSMO) study showed that about a third of children suffered from mental health problems according to the Strengths and Difficulties Questionnaire (SDQ) proxy-report by their parents (17). This was again higher than pre-pandemic levels while no significant differences were observed between different waves.

An additional stressing factor for children during the pandemic might stem from their parents, who themselves have been highly burdened at the same time, posing another risk factor for children's wellbeing (18). The mental health of parents and of their children are closely intertwined and the risk of violence against children increases through a burdened family environment (19). Against this backdrop, it is particularly important to monitor children's health-related QoL and psychological status and to identify how they can be strengthened and protected during the present and future crises.

Our study, a sub-study embedded in the larger COVID Kids Bavaria study, thus sought to analyze changes in behavior and health-related quality of life of children attending elementary schools and daycare facilities in Bavaria, Germany in spring 2021. More specifically, we investigated the following aspects: (a) behavior change during the pandemic compared to pre-pandemic behavior, (b) health-related QoL compared to pre-pandemic reference values among a nationally representative sample of healthy children, (c) health-related QoL comparing children's self-assessment with their parents' assessment, (d) determining factors for health inequalities in children during the pandemic, depicted as differences in QoL.

We hypothesized that children in our sample would demonstrate decreased health-related QoL as well as altered, presumably negatively, changed behavior patterns. We also hypothesized that children of different socioeconomic backgrounds would be differentially affected. The COVID Kids Bavaria main study question investigated the occurrence of asymptomatic cases of SARS-CoV-2 infections among children and staff in daycare facilities and elementary schools in Bavaria. Results of this SARS-CoV-2 infection surveillance were previously published (20).

## 2. Methods

COVID Kids Bavaria is a multi-center, open cohort study covering every electoral district in Bavaria, Germany. It was registered with the German Clinical Trials Register (http://www.drks.de/DRKS00022380) and approved by six local ethics committees. Methods described in this report focus on the survey component of the COVID Kids Bavaria study, whereas methods of the SARS-CoV-2 infection surveillance component of the study are reported elsewhere (20).

### 2.1. Population

Six study centers (Augsburg, Erlangen, Regensburg, Würzburg, Technische Universität München, Ludwig-Maximilians-Universität München) collected data in all 46 Bavarian electoral districts with selected facilities evenly distributed among them (20). Initially 149 facilities were enrolled (101 childcare facilities and 49 elementary schools) in September 2020. 147 facilities were visited by study teams. Towards the end of the study additional 20 elementary schools were recruited for administering questionnaires to further increase the study population. The regional distribution of the participating institutions is displayed in Supplementary Figure S1.

Participating families were eligible for enrollment if they met the following inclusion criteria: child attending the enrolled schools or daycare facilities (aged between 1 and 10 years) and written informed consent provided by parents or legal representatives. Potential participants were approached from September 2020 up to May 2021 in three consecutive phases. Eligibility to participate in the QoL KINDL^R^ questionnaire was granted for the age group of 3 years and older.

### 2.2. Data collection

At selected elementary schools and childcare facilities, an information letter about the study was distributed to all families. After providing written informed consent, parents received a personal access link to the web-based, electronic questionnaire *via* email. Online forms were pseudonymized during data entry.

Great care was taken by the consortium to ensure the comprehensibility of the questionnaire. Thus, the focus was put on the use of simple language for lay participants with the aim to generate understandable information material. Also, the questionnaire offered free field comments to point out unclear aspects of the survey. In addition, a pilot phase was conducted in July 2020 in which the questionnaire was tested and adapted accordingly. The KINDL^R^ represents a validated questionnaire, previously tested for comprehensibility in the respective target groups.

Extrapolating from the size and number of enrolled facilities, we expected 15 827 families with school children and 8,586 families with preschool children to be eligible. Of these, 3,166 families with children (1,891 families with school children and 1,275 families with preschool children) consented to participate in the study and to fill in an online questionnaire.

The questionnaire contained socioeconomic variables, demographics, measures taken in regard to hygiene and mitigation, behavior change, the parent or caretaker situation and external contextual factors.

The customized database for electronic data capture was programmed with the Castor software that is compliant with regulations such as ICH E6 Good Clinical Practice, GDPR, HIPAA, FDA 21 CFR Part 11, ISO 27001, and ISO 9001 [Castor Electronic Data Capture (Castor EDC, Amsterdam, the Netherlands)].

### 2.3. Operationalization of outcomes and variables

We assessed person-related covariates, such as the children's sex and the type of institution (elementary school, kindergarten, nursery, combined institution). Type of institution was used as an indicator for the child's age group as proxy. We defined migration background as either the child itself or at least one parent indicating a country of origin other than Germany. The family structure as well as the number of children living in the household were directly reported by the parents. Parental education was assessed based on years of education of whichever parent answered the questionnaire and then categorized into three groups (≤9 years, ≤12 years, >12 years). In addition, the children's parents were divided into system-relevant and non-system-relevant “workers”, with system-relevant professions regarded as essential for the continuation of essential services, such as supermarkets, schools and healthcare settings.

Changes in behavior were assessed by parents' report on the following topics: eating behavior, sleeping behavior, sports, outdoor activities, meeting friends offline, meeting friends online, screen time (leisure), screen time (education). Parents rated the changes in behaviour (more than in pre-pandemic times, equal, less than in pre-pandemic times) for each of the topics by comparing the one-year time period after the onset of the pandemic to pre-pandemic times.

Health-related QoL was assessed using the KINDL^R^ questionnaire. It can be answered either by the children themselves (self-report) or by their parent or caretaker (parental assessment). Depending on the children's age and developmental stage, up to four Likert-scaled questions are asked across each of six dimensions of subjective well-being (physical well-being, emotional well-being, self-esteem, family, friends, and school). The items are answered using up to five-level response categories.

The KINDL^R^ was developed by Ravens-Sieberer & Bullinger and is publicly available (https://www.kindl.org/) (21, 22). It has been used in different international health contexts including by the nationally representative KiGGS/BELLA study (the mental health module of the German Health Interview and Examination Survey for Children and Adolescents (KiGGS) initiated by the Robert Koch Institute (RKI)) (21, 23–30). Three versions of the KINDL^R^ are available comprising 12 or 24 questions (as self-report and parental proxy-report), depending on the children's age. In this study, the Kiddy KINDL^R^ version for children was used for children aged 3–6 years, as well as the Kid KINDL^R^ version for children aged 7–10 years. Parents answered the corresponding KINDL^R^ caretaker version. It should be noted, however, that the child-assessed Kiddy KINDL^R^ is validated only for the age group 4–6 years, whereas the parent-assessed version is validated for the age group 3–6 years. While the parent-assessed (proxy) versions are identical for both KINDL^R^ versions and together with the self-reported Kid KINDL^R^ consist of 24 items that can be translated into 6 sub-scores and one total score, the Kiddy KINDL^R^ version differs in its structure. Due to the challenge of interviewing very young children, the self-reported version's total score has only 12 items, 2 items for each of the 6 dimensions. Therefore, it is not possible to calculate any sub-scores for this KINDL^R^ version. Another difference of this version lies in the three-level response categories, which range from 1 = “never“ to 3 = “quite often“ in contrast to five-level response categories for children aged 7–10 (1 = “never” to 5 = “always”). A visual overview of the different KINDL^R^ versions as well as their respective sub-scores and dimensions used in this report can be found in the appendix (Supplementary Figure S2).

According to the KINDL^R^ manual the total score and six sub-scores are calculated independently for the KINDL^R^ and the Kid KINDL^R^ (31). The responses per scale are added up, whereby certain items need to be negatively recoded and pooled beforehand, creating a mean value substitution (“sum score”). Any scale can only be evaluated if no more than 30% of its items are missing (corresponding to max. 7 for the total score and 1 for any sub-scale, respectively). The resulting scores are then transformed onto a scale ranging from 0 to 100, representing the respective health-related QoL in any given dimension (e.g., Family, Friends, School, or Total score), with a higher value indicating a better QoL (see Supplementary Figure S2).

Parent-reported KINDL^R^ scores were additionally analysed in relation to reported changes in behavior.

### 2.4. Data analysis

For our analysis, we included data of questionnaires completed from April to May 2021 one year after the onset of the COVID-19 pandemic in Germany. A respective flowchart depicts the inclusion of participants (Supplementary Figure S3).

Descriptive statistics include the person-related key characteristics described above (age and sex of children, household size and migration background of family) as well as behavior change during the COVID-19 pandemic compared to pre-pandemic times. Missing values were kept as missing.

For the health-related QoL in children, means and standard deviation of the KINDL^R^ total and sub-scores were compared to pre-pandemic values using population averages from the German KiGGS/BELLA study. For this part, we distinguished between the age groups 3–6 years and 7–10 years.

Logistic regressions were undertaken to identify factors affecting children's health-related QoL during the COVID-19 pandemic. The outcome variable was defined as a lower total score on the KINDL^R^ questionnaire compared to the median of the respective population averages (median split). For the univariate as well as the multivariate regression, the two parent-assessed KINDL^R^ reports (age categories 3–6 and 7–10) were dichotomized (*via* median split) and then pooled (“health-related QoL better than median” vs. “health-related QoL worse than median”). The same pooling strategy was applied to the child-assessed KINDL^R^ reports of both age categories. The covariates are shown in Table 1 and describe the socioeconomic background of the families, the daily life of the children during the pandemic, and information regarding the parents' occupation.

**Table 1:** Characteristics of the analyzed COVID Kids Bavaria study sample one year after the onset of the pandemic in spring 2021 (*N* = 1,962).

	*N*	%
**Setting**		
Children in elementary schools	1,244	63.4%
Children in daycare facilities	718	36.6%
**Sex**		
Male	1,023	52.1%
Female	935	47.7%
Diverse	1	0.1%
Missing	3	0.2%
**Migration background**		
No	1,611	82.1%
Yes	340	17.3%
Missing	11	0.6%
**Family structure**		
Single parent	99	5.0%
Single parent and partner	38	1.9%
Both parents in household	1,807	92.1%
Changing responsibilities	13	0.7%
Other modell	5	0.3%
**Number of children in household**		
1	396	20.2%
2	1,044	53.2%
3	330	16.8%
>3	47	2.6%
Missing	145	7.4%
**System relevant parental occupation**		
No	1,172	59.7%
Yes	790	40.3%
**Years of paternal education**		
≤9 Years	83	0.0%
10–12 Years	132	0.1%
>12 Years	1,080	55.0%
Missing	667	34.0%
**Years of maternal education**		
≤9 Years	71	0.0%
10–12 Years	88	0.0%
>12 Years	1,136	57.9%
Missing	667	34.0%

Children's self-assessed KINDL^R^ scores were contrasted with parent-assessed KINDL^R^ scores *via* a t-test for paired samples and Cohen's kappa to test the inter-rater reliability of the two assessments.

As an exploratory approach to observe the effect of various adaptive behavior patterns during the pandemic, we descriptively compared the KINDL^R^ scores (means and standard deviations) stratified by the different types of behavior change.

Univariate logistic regression analyses with pooled KINDL^R^ scores as outcome variables (median split) were performed and corresponding ORs (odds ratios) and 95% CIs (confidence intervals) were reported.

All analyses were undertaken in IBM SPSS Statistics 27.

## 3. Results

### 3.1. Descriptive characteristics

Comparing the expected number of participants with the actual number of participants shows a response rate of 12.9% (11.9% for school children, 14.8% for pre-schoolers) over the entire study period. We included 1962 child-parent-pairs with completed questionnaires (Table 1). Participants and non-participants were shown to be similar with regards to demographics and educational level, as reported in the main study paper by Kern et al. (20).

The majority of children attended Bavarian elementary schools (*n* = 1244, 63.4%) while 36.6% of the children (*n* = 718) attended childcare centers. Sex distribution was balanced (boys 52.1% vs. girls 47.7%, with one child specified as diverse). 17.3% of children had at least one parent born outside of Germany and 40.3% of children had a parent engaged in a profession considered “system relevant” during the COVID-19 pandemic. A detailed overview of the distribution concerning the children's living conditions and family-related covariates can be found in Table 1.

In addition, we used variables to assess the family impact of the COVID-19 pandemic. Thus, our data showed that 4.5% of the respondents themselves and 2.3% of their partners were no longer working due to the pandemic. Regarding healthcare access during the pandemic, 5.4% of parents reported refraining from visiting a doctor with their child due to the pandemic. While 65.5% of all parents reported that all medical appointments took place, 6.2% reported that their children's medical appointments were cancelled during these times.

Concerning attitudes toward vaccination, 71.7% expressed interest in COVID-19 vaccination for their children which was not available at the time of questionnaire distribution. In total, 87.5% of included children were fully vaccinated against childhood infectious diseases according to the German Standing Committee on Vaccination (STIKO) recommendations while 3.7% were not fully vaccinated. In comparison to previously published vaccination rates, the rates in our sample are high (32).

### 3.2. Changes in behavior compared to pre-pandemic times and in relation to health-related quality of life

Parents were asked to judge their children's behavior patterns compared to pre-pandemic times. Regarding sport/ physical activity, the majority reported that their child did less sport. General screen time for leisure and education purposes increased, while time with friends was also reportedly to shift towards more online time. Parents also observed considerable changes concerning the eating and sleeping behavior of their children, with changes in both directions, indicating maladaptive coping strategies in each case. A detailed overview is displayed in Table 2.

**Table 2:** Subjective changes in behavior one year after the onset of the pandemic compared to pre-pandemic times assessed by parents (*N* = 1,962).

	Eating behavior	Sleeping behavior	Sports	Outdoor activities	Meeting friends offline	Meeting friends online	Screen time (leisure time)	Screen time (education)
	*n (%)*	*n (%)*	*n (%)*	*n (%)*	*n (%)*	*n (%)*	*n (%)*	*n (%)*
Total *N*	***1*,*962***	***1*,*962***	***1*,*962***	***1*,*962***	***1*,*962***	***1*,*962***	***1*,*962***	***1*,*962***
More than in prepandemic times	247 (12.6%)	144 (7.3%)	77 (3.9%)	407 (20.7%)	39 (2.0%)	573 (29.2%)	1,289 (65.7%)	1,200 (61.2%)
Equal	1,580 (80.5%)	1,402 (71.5%)	658 (33.5%)	976 (49.7%)	175 (8.9%)	910 (46.4%)	633 (32.3%)	218 (11.1%)
Less than in prepandemic times	118 (6.0%)	399 (20.3%)	1,220 (62.2%)	569 (29.0%)	1,739 (88.6%)	322 (16.4%)	21 (1.1%)	16 (0.8%)
Don't know	17 (0.9%)	17 (0.9%)	7 (0.0%)	10 (0.5%)	9 (0.5%)	157 (8.0%)	19 (1.0%)	15 (0.8%)
Not applicable	0 (0.0%)	0 (0.0%)	0 (0.0%)	0 (0.0%)	0 (0.0%)	0 (0.0%)	0 (0.0%)	513 (26.1%)

Comparing the parent-assessed KINDL^R^ scores associated with different changes in behavior, we observed that results were similar for both age categories. Therefore, for the sake of clarity, this paragraph will always refer to the KINDL^R^ total score of the 7–10-year-olds. All results for all age categories are displayed in Supplementary Table A1.

Eating more and eating less than before the pandemic were both associated with worse KINDL^R^ total scores (eating more: MD 67.40 ± 12.76); eating less: MD 64.05 ± 13.18) compared to children whose eating behavior had not changed during the pandemic according to their parents' judgement (MD 76.04 ± 10.87). Similarly, for sleep behavior, the children who experienced changes in behavior (more or less sleep than before the pandemic) also had a clearly worse KINDL^R^ total score than their peers whose sleep behavior had remained the same (MD 76.56 ± 10.62), with the children who slept less than before having even worse KINDL^R^ scores (MD 66.91 ± 12.97) than the children who slept more than before (MD 73.34 ± 11.70). Although few in number (*n* = 77), children who did more sport during the pandemic showed a higher KINDL^R^ score (MD 80.93 ± 9.11) than children with unchanged sports behavior (MD 78.93 (±10.49) and especially children who played less sports during the pandemic (MD 72.13 ± 12.05). Children whose leisure time remained unchanged compared to pre-pandemic times reported better KINDL^R^ scores (MD 78.00 ± 10.94) than children who spent more time in front of a screen during the pandemic (MD 72.16 ± 12.07). This would also apply to children whose screen time decreased during the pandemic, but numbers were small (*n* = 16).

### 3.3. Health-related QoL measured with KINDL^R^ scores compared to pre-pandemic population averages

We compared the parent-assessed KINDL^R^ of the COVID Kids Bavaria cohort one year after the onset of the pandemic to pre-pandemic reference values provided by the German KiGGS/BELLA cohort, a longitudinal study on the health of children and adolescents in Germany conducted between 2003 and 2006 (Table 3) (30, 33).

**Table 3:** Health-related quality of life (QoL) measured with the KINDL^R^ one year after the onset of the pandemic compared to reference data in healthy population (30, 33).

KINDL^R^ scores	Parent-assessed KINDL^R^ score (Kids aged 3–6)		Prepandemic reference	Parent-assessed KINDL^R^ score (Kids aged 7–10)		Prepandemic reference
	*N*	M (SD)	M (SD)	*N*	M (SD)	M (SD)
KINDL^R^—Total score	633	74.78 (10.57)	80.00 (8.10)	1,034	73.88 (12.03)	79.30 (9.00)
KINDL^R^—Physical		82.00 (14.60)	80.20 (15.70)		78.76 (16.26)	80.60 (16.30)
KINDL^R^—Emotional		74.75 (15.62)	83.00 (11.40)		71.81 (17.31)	82.70 (12.10)
KINDL^R^—Self-Esteem		73.80 (13.20)	73.60 (13.30)		69.69 (15.25)	71.40 (13.20)
KINDL^R^—Family		74.89 (13.52)	80.70 (11.90)		74.86 (14.77)	79.90 (12.60)
KINDL^R^—Friends		68.62 (15.86)	79.70 (12.30)		71.33 (15.86)	78.50 (13.00)
KINDL^R^—School		74.69 (20.85)	83.80 (12.50)		77.00 (17.59)	83.10 (14.20)

Higher score values indicate higher QoL.

In general, we observed lower total scores in all KINDL^R^ analyses in the COVID Kids Bavaria cohort compared to pre-pandemic population averages across all age groups (for 3–6-year-old KINDL^R^-total score: COVID Kids Bavaria MD 74.78 ± 10.57 vs. KiGGS data 80.0 ± 8.1; 7–10 years-old KINDL^R^-total score: COVID Kids Bavaria MD 73.88 ± 12.03 vs. KiGGS data 79.30 ± 9.0).

Regarding the KINDL^R^ sub-scores, Emotional Well-Being, Family, Friends, and School, the KINDL^R^ scores for all age groups were lower one year after the onset of the pandemic compared to pre-pandemic population averages. Also, in the dimensions of Physical Well-Being (COVID Kids Bavaria MD 78.76 ± 16.26 vs. KiGGS data 80.60 ± 16.3) and Self-Esteem (COVID Kids Bavaria MD 69.69 ± 15.25 vs. KiGGS data 71.40 ± 13.2) lower KINDL^R^ values were found for the 7–10-year-olds. In contrast, the values for the 3–6-year-olds were slightly above pre-pandemic population averages in these dimensions (Physical Well-Being: COVID Kids Bavaria MD 82.00 ± 14.6 vs. KiGGS data 80.20 ± 15.7; Self-esteem: COVID Kids Bavaria MD 73.80 ± 13.2 vs. KiGGS 73.60 ± 13.3).

Comparing the parent-assessed KINDL^R^ scores to the children's self-reports, our results indicate that they do not correspond well, with the t-test also indicating a significant difference between parental and child assessment (Supplementary Table A2, A3). The kappa-values for interrater-reliability shown in these tables generally translate to no more than a slight strength of agreement (34).

Across all age groups, children's self-report on health-related QoL was significantly higher than the parent-assessed QoL, indicating that children consistently assessed their situation more positively than their parents, with only one exception for self-esteem, where the 7–10 year old children rated their QoL slightly worse than their parents. Our findings seem to be stable over time, as they align with the KINDL^R^ scores of 2068 children assessed from November 2020 – March 2021 who display similar KINDL^R^ outcomes compared to the time period analyzed by us (April-May 2021) (Supplementary Table A4).

### 3.4. Equity considerations in health-related QoL (univariate and multivariate analyses)

We sought to identify subgroups that may be especially vulnerable to a worsening of health-related QoL during the pandemic, thereby indicating a health disadvantage. To do so we performed binary univariate regression analyses with a pooled outcome variable of the KINDL^R^ score, combining all age categories into a dichotomized median split.

As shown in Table 4, we did not find any differences between those children with a high or low health-related QoL (median split) for the covariates type of institution, sex, migration background, family structure, household size, system relevant parental occupation and parental education, for the parent-assessed KINDL^R^ report for children aged 3–10 years. However, with the children-assessed KINDL^R^ report, our results indicate that boys were less likely to report a KINDL^R^ total score above the sample's median compared to girls (OR: 0.82 [0.68; 0.99]). Regarding the family concept, children living with both parents in one household were more likely to experience an above-median health-related QoL (KINDL^R^ total score) compared to children living with a single parent (OR: 1.62 [1.03; 2.55]).

**Table 4:** Univariate logistic regression (median split of parent-assessed and children-assessed health-related quality of life) for children aged 3–10.

Likelihood of a health-related Quality of Life score above the Median (all age groups pooled together)				
	Parent-assessed KINDL^R^ (all age groups pooled together)		Children-assessed KINDL^R^ (all age groups pooled together)	
	% of participants with KINDL^R^ score <median	OR [95% CI]	% of participants with KINDL^R^ scores <median	OR [95% CI]
**Type of institution**				
Primary School (Ref.)	47.1%		47.9%	
Kindergarden	47.6%	1.02 [0.76; 1.37]	49.7%	0.79 [0.61; 1.03]
Nursery	50.0%	1.12 [0.53; 2.38]	60.0%	0.85 [0.59; 1.22]
Combined institution	51.0%	1.17 [0.91; 1.50]	53.8%	1.29 [0.56; 2.96]
**Sex**				
Female (Ref.)	50.0%		51.9%	
Male	46.0%	0.85 [0.70; 1.03]	47.0%	**0.82 [0.68; 0.99]** *
**Migration background**				
No (Ref.)	48.2%		49.9%	
Yes	46.8%	0.95 [0.73; 1.22]	47.1%	0.90 [0.69; 1.16]
**Family structure**				
Single parent (Ref.)	37.8%		38.6%	
Single parent and partner	28.6%	0.66 [0.28; 1.55]	26.5%	0.57 [0.24; 1.40]
Both parents in household	48.7%	1.56 [0.99; 2.47]	50.4%	**1.62 [1.03; 2.55]** *
Changing responsibilities	61.5%	2.63 [0.79; 8.77]	53.8%	1.86 [0.58; 6.03]
Other model	75.0%	4.94 [0.49; 49.56]	75.0%	4.78 [0.48; 47.97]
**Number of children living in the household**				
1 (Ref.)	47.2%		49.0%	
2	49.2%	1.08 [0.83; 1.40]	50.4%	1.06 [0.81; 1.38]
3	49.1%	1.08 [0.78; 1.49]	51.1%	1.09 [0.78; 1.51]
>3	60.5%	1.71 [0.89; 3.29]	57.5%	1.41 [0.72; 2.75]
**System relevant parental occupation**				
No (Ref.)	48.1%		50.0%	
Yes	47.7%	0.99 [0.83; 1.40]	48.4%	0.94 [0.77; 1.15]
**Parental education**				
≤9 years (Ref.)	42.9%		47.4%	
10–12 years	46.2%	1.15 [0.64; 2.04]	46.5%	0.97 [0.54; 1.73]
>12 years	48.5%	1.26 [0.79; 2.00]	50.0%	1.11 [0.70; 1.76]

CI: Confidence Interval.

*indicating a *p*-value ≤0.05; bold font indicating significance.

Under the same assumptions, we ran multivariate binary regressions for both the pooled parent-assessed outcome and the child-assessed outcome (Table 5). For both KINDL^R^ outcomes, we could not detect any significant differences between the children aged 3–10 years with above-median or below-median health-related QoL considering the mostly socio-economic covariates described above.

**Table 5:** Multivariate logistic regression with health-related QoL outcome (median split) for children aged 3–10.

Likelihood of a health-related Quality of Life score above the Median (all age groups pooled together)		
	Parent-assessed KINDL^R^	Children-assessed KINDL^R^
	OR [95% CI]	OR [95% CI]
**Type of institution**		
Primary School (Ref.)		
Kindergarden	0.99 [0.72; 1.37]	1.05 [0.75; 1.46]
Nursery	1.13 [0.52; 2.46]	1.51 [0.66; 3.44]
Combined institution	1.11 [0.84; 1.46]	1.15 [0.87; 1.53]
**Sex**		
Female (Ref.)		
Male	0.86 [0.70; 1.06]	0.83 [0.67; 1.02]
**Migration Background**		
No (Ref.)		
Yes	0.95 [0.72; 1.25]	0.97 [0.73; 1.28]
**Family structure**		
Single parent (Ref.)		
Single parent and partner	0.53 [0.21; 1.35]	0.38 [0.14; 1.03]
Both parents in household	1.21 [0.72; 2.01]	1.21 [0.73; 2.01]
Changing responsibilities	2.41 [0.65; 8.91]	1.59 [0.45; 5.61]
Other model	4.16 [0.41; 42.48]	3.76 [0.37; 38.25]
**Number of children living in the household**		
1 (Ref.)		
2	1.06 [0.80; 1.39]	1.03 [0.78; 1.36]
3	1.07 [0.76; 1.50]	1.06 [0.75; 1.50]
>3	1.80 [0.91; 3.56]	1.34 [0.67; 2.69]
**System relevant parental occupation**		
No (Ref.)		
Yes	0.98 [0.78; 1.23]	0.95 [0.75; 1.20]
**Parental education**		
≤9 years (Ref.)		
10–12 years	1.07 [0.59; 1.96]	1.02 [0.56; 1.90]
>12 years	1.14 [0.71; 1.84]	1.07 [0.66; 1.73]
**Pseudo R^2^**	0.014	0.017

CI: Confidence Interval.

* indicating a *p*-value ≤0.05; bold font indicating significance.

## 4. Discussion

The COVID Kids Bavaria study assessed the incidence of SARS-CoV-2 and its ramifications in healthy children attending elementary schools and day care facilities in different phases of the COVID-19 pandemic. Herein, we investigated health-related QoL and changes in behavior of children with a focus on the time point one year after the onset of the COVID-19 pandemic.

Main results were that a high percentage of parents reported pronounced changes in behaviour in their children, which were found to be associated with reduced health-related QoL. Children's health-related QoL was lower one year after the onset of the pandemic compared to pre-pandemic levels while children still had a more positive QoL perception than their parents.

These findings are in line with other studies reporting a deterioration in wellbeing and mental health among the youngest during the COVID-19 pandemic nationally (15) and internationally (35–40). Prior to the pandemic, in 2017, 17.2% of children and adolescents aged 3 to 17 years in the German BELLA cohort study showed psychosocial problems measured with the SDQ (41). Similarly, a meta-analysis from 2012 reported that about one in five children were at risk of mental health problems (42) while recent publications now report one in three children to be at risk (17). With regard to KINDL^R^-specific values, a German study from 2016 showed that the KINDL^R^ scores and therefore the health-related QoL perception of children and parents alike had increased consistently over the past 10 years before the start of the COVID-19 pandemic (43). This would point towards an even higher difference compared to pre-pandemic values.

Overall, children aged 3–10 years in our cohort had much more positive perception of their current QoL than their parents, with children generally reporting higher KINDL^R^ total scores and sub-scores. This, however, is in contrast to pre-pandemic studies comparing parents' and children's KINDL^R^ reports, where the picture is less consistent. In a German study comparing parents' and children's KINDL^R^ reports stemming from the KiGGS study (from 2003 to 2006), parents significantly overestimated their children's health-related QoL, both in the KINDL^R^ total score and in the sub-scores for the dimensions Physical Well-Being, Self-esteem, and School. At the same time, they underestimated the sub-scores Emotional Well-Being and Family (24, 44). For a direct interpretation, however, it is important to know that the children considered in the KiGGS cohort were older than in our sample, namely 11–17 years old. Similar results were also reported in a Norwegian study from 1999, in which parents generally reported significantly more positive KINDL^R^ scores than their 9–16-year-old children (45). Overall, our results suggest that the COVID-19 pandemic might have exacerbated pre-existing discrepancies in the perceptions of children's QoL, making it even more difficult for parents to assess their children's actual situation. Alternatively, due to the high stress levels during this situation (18), parents could have tried to compensate this by being especially concerned about their children, resulting in evaluating their QoL worse than it actually was. The QoL tends to deteriorate with the age of the children. One could also deduce that especially younger age groups in our sample perceive the threatening situation differently from their parents.

Regarding changes in behavior, nearly 20% of this selected cohort ate either more or less than before the pandemic, 30% changed their sleeping behavior, 60% of children engaged in fewer sports activities than before, and 65% of children spent more leisure time in front of a screen. These results are in line with other international studies. Thus, trajectories in psychosocial aspects have already been described in the literature, all of them reported changes in children's and adolescents' sleeping behavior, reductions in physical activity and increases in screen time (46–51). On this basis, a 2020 US study recognized the positive association between higher levels of physical activity or less screen time and better mental health outcomes during the pandemic, as measured by the SDQ (52). Furthermore, a Turkish study of children during the pandemic showed the association between increased Internet use and worsened KINDL^R^ scores (48).

A systematic review also stressed the association of poor mental health with sleeping problems, which could lead to a downward trajectory during the COVID-19 pandemic and could partially explain the study results (53). Screen time also tends to be related to sleeping problems and sleeping deviations, which—as shown by our data—has increased during the COVID-19 pandemic, both school-related and during leisure time. Thus, the observed changes in behavior and associated KINDL^R^ scores may also be interdependent to some extent.

Univariate analyses of the children self-reported KINDL^R^ revealed that being male was a risk factor for a below-median health-related QoL, whereas living in a household with both parents increased the likelihood of an above-median QoL. However, based on our multivariate analyses of parent-assessed and children-assessed KINDL^R^ outcomes, we were not able to identify any predicting factor for above- or below-median health-related QoL that could indicate a specific vulnerability for health inequalities during the pandemic.

In contrast, published evidence often reports female sex as a risk factor for poor mental health during the COVID-19 pandemic, as it is also considered a risk factor for depression in general (54–58).

### 4.1. Limitations

The overall low response rate limits the external validity of the study with a high likelihood for systematic bias. The results shown here should thus be interpreted with caution due to the high probability of a selection and non-responder bias in our study sample. Thus, data cannot be considered to be representative of Bavarian children as a whole. Our study cohort rather provides information about which population groups and segments are willing to participate in a questionnaire-based study in a school setting during a pandemic crisis.

Based on a non-responder analysis for COVID Kids Bavaria (20), the characteristics of the responders of this convenience sample shows that a systematic middle-class bias is likely to be present. For instance, 17.3% of all children in our cohort have a migration background, whereas the Bavaria-wide share of families with a migration background was 34.2% in 2019 and is thus strongly underrepresented in our sample and may explain why our covariate analysis for migration background did not reveal any differences for a high or low health-related QoL (median split) (59). The situation is similar for single parent status: while 15.1% of all families across Bavaria were single parents in 2019 (59), the share in our cohort is only 6.9%. The number of children per household in our sample also differs from the Bavarian average: in 2019, 49.5% of households with children had one child, 39.1% had two children, and 11.4% had three or more children (59). Our sample overestimates the number of households with two children (53.0% instead of 39.1%) and underestimates the number of one-child households (20.0% instead of 49.5%).

Consequently, the families in our sample have less migration background, are more likely to be married and less likely to be single parents, and are more likely to have two children. Notably, these factors were also found to be generally protective against health inequalities, potentially leading to distortions in our data that may mask determinants of health disadvantage.

Based on the characteristics of our sample and the comparison with the Bavarian general population, our participants seem to come from the rather well-off middle class milieu and are therefore not representative for Bavaria as a whole. Other possible homogeneities in the cohort, such as common beliefs or motivation, may not be traceable at all. A particularly strong bias is also evident in the years of parental education: for both fathers and mothers, 34.0% did not indicate any answer. While the percentage of missing values was relatively low for most variables, the variables for paternal and maternal education both showed 34.0% of missing values, which could very likely distort the picture. Thus, our results have only a limited external validity and are therefore only transferable to Bavarian primary school and daycare facility children to some extent.

Self-assessment may represent another source of bias, potentially leading to recall bias, optimism bias, or social-desirability bias. However, as results point in a similar direction as parental external assessments and evidence from other studies, we believe that this effect should not be overestimated.

### 4.2. Implications for research and practice

A growing body of evidence shows that children suffer from significant psychosocial consequences during this pandemic. However, it remains a challenge to differentiate the effects of acute and chronic somatic, psychological and social sequelae of COVID-19 and its mitigation measures in individual children and pediatric populations as a whole. Therefore, further analyses in large-scale longitudinal studies and surveillance data are needed to understand how the various factors affect quality of life, changes in behavior and mental health in children. For future crises and pandemics, policymakers, researchers and clinicians should prioritize the wellbeing, psychosocial monitoring and mental health right from the beginning to identify and protect vulnerable groups, to identify targets for interventions for those at risk and to promote mental health and resilience. As it now became evident that the pandemic affects all areas of children's lives, these efforts should not only be limited to mitigation measures but also other pandemic aspects. The approach of the German Wü-KiTa-CoV-Project on the feasibility of SARS-CoV-2 testing in daycare centers gives an example of how the situation of children could not only be well monitored, but also of how to include the children's perspective through qualitative work and how to test different models for improvement through intervention studies (60–62).

In terms of policies and practice, widespread awareness, research and implementation of evidence based (mental) health protection and promotion efforts in children during crises should be undertaken. While at the same time inequalities should be balanced through focus on the disadvantaged by drawing on international and national guidance (63, 64).

## Data Availability

Inquiries regarding the raw data supporting the conclusions of this study can be directed to the corresponding authors.
