# Supplementary material for: Changes in behavior and quality of life in German young children during the COVID-19 pandemic—results from the COVID kids bavaria study
Source: Front Pediatr. 2023 May 9;11:1135415. doi: 10.3389/fped.2023.1135415 (PMC10204608; doi:10.3389/fped.2023.1135415)
Supplement: Supplementary file 1 [file Datasheet1.docx]

Supplementary Material

# Supplementary Figures and Tables

## Supplementary Figures

**Supplementary Figure S1.** Participating institutions of this substudy on a Bavarian map.


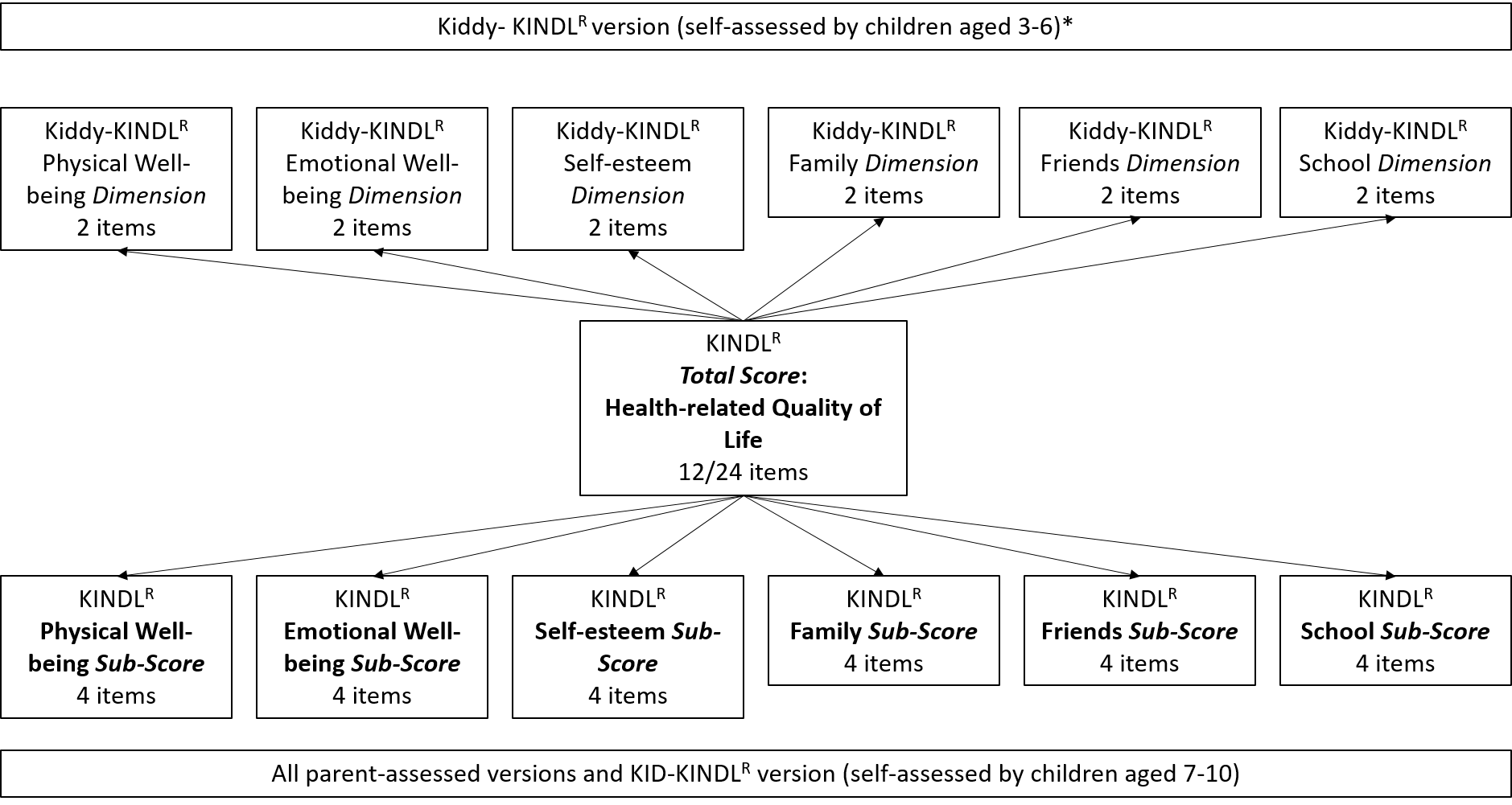


**Supplementary Figure S2.** Overview of all applied KINDL^R^ versions and dimensions that we applied.


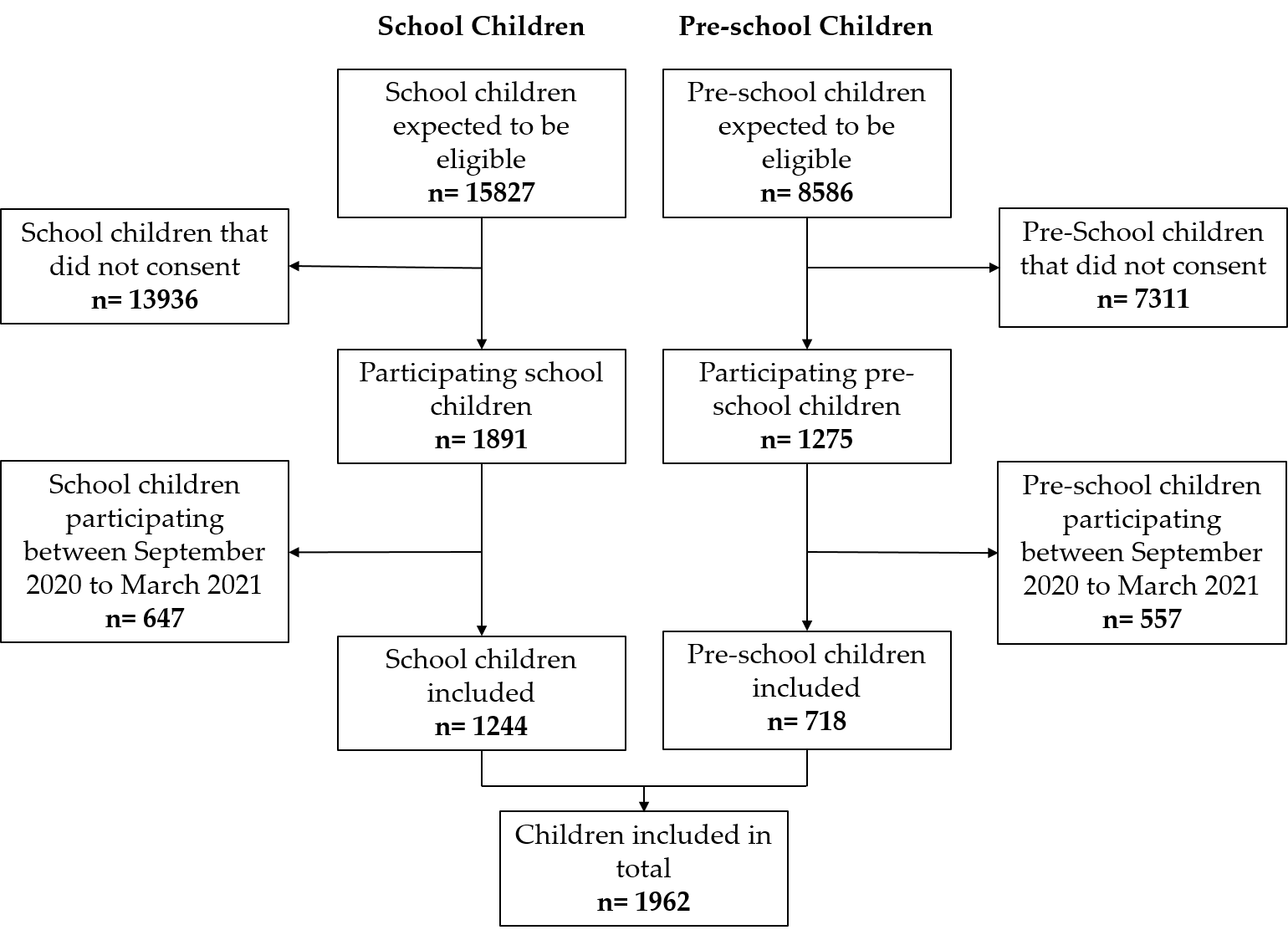


**Supplementary Figure S3.** Participant flow-chart.

## Supplementary Tables

|  | **Eating behavior** | | **Sleeping behavior** | | **Sports / physical activity** | | **Screen time (leisure)** | |
| --- | --- | --- | --- | --- | --- | --- | --- | --- |
| **behavior change during the COVID-19 pandemic** *(assessed between April-May 2021)* | parent-assessed  KINDL^R^, aged 3-6 years | parent-assessed  KINDL^R^, aged 7-10 years | parent-assessed  KINDL^R^, aged 3-6 years | parent-assessed  KINDL^R^, aged 7-10 years | parent-assessed  KINDL^R^, aged 3-6 years | parent-assessed  KINDL^R^, aged 7-10 years | parent-assessed  KINDL^R^, aged 3-6 years | parent-assessed  KINDL^R^, aged 7-10 years |
|  | M (SD) | M (SD) | M (SD) | M (SD) | M (SD) | M (SD) | M (SD) | M (SD) |
| **more than prepandemic times** | 68.45 (11.51) | 67.40 (12.76) | 71.10 (9.32) | 73.34 (11.70) | 76.47 (8.66) | 80.93 (9.11) | 72.88 (10.55) | 72.16 (12.07) |
| **equal** | 75.79 (10.08) | 76.04 (10.87) | 76.09 (10.54) | 76.56 (10.62) | 77.57 (9.31) | 78.93 (10.49) | 77.90 (9.60) | 78.00 (10.94) |
| **less than prepandemic times** | 68.55 (11.72) | 64.05 (13.18) | 69.51 (9.36) | 66.91 (12.97) | 72.00 (11.17) | 72.13 (12.05) | 74.34 (13.00) | 75.27 (9.74) |

**Supplementary Table A1.** KINDL^R^ in relation to behavior change.

|  | **Assessed by parents of children aged 3-6** | | **Assessed by children aged 3-6** | | **t-Test** | | **Cohen's kappa** | |
| --- | --- | --- | --- | --- | --- | --- | --- | --- |
|  | N | M (SD) | N | M (SD) | Coef. | p-value | Coef. | p-value |
| **KINDL^R^ – Total score** | 633 | 74.78 (10.57) | 604 | 78.95 (13.52) | **9.45** | 0.000 | 0.01 | 0.101 |

Note: bold font indicating significance.

Supplementary Table A2. Comparison of child- and parent-assessed KINDL^R^ score for children aged 3-6 years from April 2021-May 2021

|  | **Assessed by parents of children aged**  **7-11 years** | | **Assessed by children aged 7-11 years** | | **t-Test** | | **Cohen's kappa** | |
| --- | --- | --- | --- | --- | --- | --- | --- | --- |
| **KINDL^R^ - Quality of Life** | N | M (SD) | N | M (SD) | Coef. | p-Wert | Coef. | p-Wert |
| **KINDL^R^ –**  **Total score** | 1034 | 73.88 (12.03) | 1024 | 76.94 (11.91) | **11.823** | 0.000 | **0.024** | <0.001 |
| **KINDL^R^ –**  **Physical** | 1046 | 78.76 (16.26) | 1041 | 80.79 (16.21) | **5.518** | 0.000 | **0.169** | <0.001 |
| **KINDL^R^ –**  **Emotional** | 1042 | 71.81 (17.31) | 1037 | 75.63 (16.58) | **8.434** | 0.000 | **0.083** | <0.001 |
| **KINDL^R^ –**  **Self-esteem** | 1038 | 69.69 (15.25) | 1032 | 68.75 (18.23) | -1.848 | 0.065 | **0.115** | <0.001 |
| **KINDL^R^ –**  **Family** | 1036 | 74.86 (14.77) | 1024 | 79.28 (14.63) | **10.677** | 0.000 | **0.090** | <0.001 |
| **KINDL^R^ –**  **Friends** | 1028 | 71,33 (15,86) | 1019 | 76,45 (15,54) | **13,550** | 0,000 | **0,164** | <0.001 |
| **KINDL^R^ -**  **School** | 1019 | 77,00 (17,59) | 1011 | 80,69 (16,50) | **8,527** | 0,000 | **0,183** | <0.001 |

Note: bold font indicating significance.

**Supplementary Table A3.** Comparison of child- and parent-assessed KINDL^R^ scores for children aged 7-10 years from April 2021-May 2021


| (n=2068) | **KINDL^R^ score of children aged 3-6 years** | | | | **KINDL^R^ score of children aged 7-11 years** | | | |
| --- | --- | --- | --- | --- | --- | --- | --- | --- |
| **KINDL^R^ - Quality of Life** | **Parent-assessed** | | **Children-assessed** | | **Parent-assessed** | | **Children-assessed** | |
|  | N | M (SD) | N | M (SD) | N | M (SD) | N | M (SD) |
| **KINDL^R^ - Total score** | 970 | 77.58 (9.62) | 1099 | 80.15 (12.39) | 874 | 76.45 (10.85) | 1024 | 83.37 (15.01) |
| **KINDL^R^ - Physical** | 970 | 82.55 (13.93) | - | - | 874 | 81.82 (15.44) | 1041 | 74.17 (15.69) |
| **KINDL^R^ - Emotional** | 970 | 78.56 (14.12) | - | - | 874 | 76.10 (16.19) | 1037 | 79.78 (13.62) |
| **KINDL^R^ - Self-Esteem** | 970 | 76.22 (12.29) | - | - | 874 | 72.90 (14.73) | 1032 | 73.05 (16.72) |
| **KINDL^R^ - Family** | 970 | 76.62 (12.93) | - | - | 874 | 77.43 (14.04) | 1024 | 77.76 (14.76) |
| **KINDL^R^ - Friends** | 970 | 69.67 (15.41) | - | - | 874 | 68.71 (15.12) | 1019 | 82.38 (15.53) |
| **KINDL^R^ - School** | 970 | 81.85 (16.91) | - | - | 874 | 81.75 (15.58) | 1011 | 78.23 (10.68) |

**Supplementary Table A4.** Health-related Quality of Life measured with KINDL^R^ for 2068 children from November 2020 – March 2021 showing similar KINDL^R^ scores to the time period analyzed (April-May 2021)
